# A TCR-based Chimeric Antigen Receptor

**DOI:** 10.1038/s41598-017-11126-y

**Published:** 2017-09-06

**Authors:** Even Walseng, Hakan Köksal, Ibrahim M. Sektioglu, Anne Fåne, Gjertrud Skorstad, Gunnar Kvalheim, Gustav Gaudernack, Else Marit Inderberg, Sébastien Wälchli

**Affiliations:** 1 Experimental Immunology Branch, National Cancer Institute, National Institutes of Health, Bethesda, MD 20892 USA; 2 Department for Cellular Therapy, Department for Cancer Therapy, Oslo University Hospital Radiumhospitalet, Oslo, Norway; 3 Department for Cancer Immunology, Institute for Cancer Research, Oslo University Hospital Radiumhospitalet, Oslo, Norway; 4 Centre for Cancer Biomedicine, University of Oslo, Oslo, Norway

## Abstract

Effector T cells equipped with engineered antigen receptors specific for cancer targets have proven to be very efficient. Two methods have emerged: the Chimeric Antigen Receptors (CARs) and T-cell Receptor (TCR) redirection. Although very potent, CAR recognition is limited to membrane antigens which represent around 1% of the total proteins expressed, whereas TCRs have the advantage of targeting any peptide resulting from cellular protein degradation. However, TCRs depend on heavy signalling machinery only present in T cells which restricts the type of eligible therapeutic cells. Hence, an introduced therapeutic TCR will compete with the endogenous TCR for the signalling proteins and carries the potential risk of mixed dimer formation giving rise to a new TCR with unpredictable specificity. We have fused a soluble TCR construct to a CAR-signalling tail and named the final product TCR-CAR. We here show that, if expressed, the TCR-CAR conserved the specificity and the functionality of the original TCR. In addition, we demonstrate that TCR-CAR redirection was not restricted to T cells. Indeed, after transduction, the NK cell line NK-92 became TCR positive and reacted against pMHC target. This opens therapeutic avenues combing the killing efficiency of NK cells with the diversified target recognition of TCRs.

## Introduction

Immunotherapy connecting the power of T cells and redirecting them against tumour has in the past 5 years proven very successful and attracted considerable interest. It includes the redirection of effector cells (mainly T and NK cells) with selected antigen receptors. To date, two main redirecting agents have been developed: modified antibodies and T-cell Receptors (TCR). Antibodies, being soluble proteins, were modified into cellular receptor by (i) fusing it to resident protein transmembrane (TM) domains and (ii) adding signalling domain of known TCR signalling proteins, mainly phosphorylation sites of partners involved in signal I and II^1–3^. The composition and combination of domains linked to the single chain variable part of the antibody (scFv) are diverse and no clear road map of the most potent universal design has been drawn so far. These Chimeric Antigen Receptors (CARs) have the capacity to generate an immune synapse and trigger effector cell functions, cytokine release and target killing. After the astonishing results generated by different teams using anti-CD19 CAR for the treatment of haematological malignancies^4–7^ the use of these constructs has had a meteoric rise. New targets are presently evaluated, but the outcome, in particular when dealing with solid tumours, was not as successful as observed with the common B-cell marker CD19^8–10^. Therefore, the obvious bottleneck in CAR development is the lack of cancer-specific targets. Indeed, when introduced into T cells, CARs are limited to antigens (proteins, sugar residues) expressed on the surface of the target cells.

The second type of receptors, TCRs, is not limited to the detection of surface antigens like antibodies. Rather they were defined as “obsessed” with peptides presented on the MHC molecules, pMHC^11^. Considering that all the proteins expressed by a given cell will be degraded and loaded onto an MHC molecule, TCRs can potentially recognize the whole proteome. This represents a striking numerical advantage over CARs in terms of possible targets. In addition, TCRs can be specifically directed against a mutant variant of a protein and spare the wild type form^12^, hence the TCR can distinguish cancer cells expressing the mutated protein from healthy cells expressing the non-mutated protein. On the other hand, TCRs are complicated molecules to manipulate: they are heterodimers composed of an α- and a β-chain, they do not signal by themselves but require a battery of signalling proteins associated to recruit all the components to create an immune synapse. In addition, their localization at the plasma membrane depends on the CD3 complex, whose expression is restricted to T cells. Consequently TCR-based redirection has only been available in T cells since they are the only cells that possess all components required for proper TCR stimulation. In addition, the exogenous TCR might compete with the endogenous TCR for the use of these signalling proteins^13^. Another issue with the introduction of a second TCR into the redirected T cell is the possibility to form mixed dimers thus generating novel TCRs^14, 15^. Although mispairing of TCRs has yet to be observed in a clinical setting, an important number of innovations has been developed in order to prevent this. The addition of extra cysteines on the constant part of both chains represented the first step to support the pairing of the redirecting TCR^16, 17^. Another strategy was to replace the constant domains of the therapeutic TCRs with murine constant domains^18, 19^. The rationale behind this was (i) mouse TCR constant domain has higher affinity to human CD3 than human constant domain^20^ and (ii) this would increase the chance of the correct TCRs pairing, accepting *per se* that xenogenous pairing would not occur. However, to our knowledge mouse and human constant parts have never been shown not to pair. Although these modifications might improve TCR expression and signalling of certain TCRs, but not universally^17–19^, one cannot exclude that the higher affinity of mouse TCR constant domain for the human CD3 could be the main mechanism behind this improved effect^20^. Thus the CD3 monopolization seems to represent the major factor improving TCR redirection observed with murinized constructs. It is worth mentioning that the use of murine protein domain in a therapeutic construct might lead to rejection by the patient’s immune system as previously reported with a non-humanized CAR^21^. Finally, another strategy to improve redirected TCR potency and avoiding the mispairing was by fusing of signalling components to the intracellular domain of one of the TCR chains^22^.

In the current study, we have generated a single chain membrane bound TCR built on a technique we validated to produce soluble TCRs (sTCR)^23, 24^. We previously demonstrated that a stable molecule could be obtained by expressing the extracellular domains of the two TCR chains linked with a 2A ribosome skipping sequence^24^. Given that the construct was efficiently produced, we reasoned that a similar stability could be generated when the sTCR was associated to the cell surface. The present construct consists of our previously published sTCR construct^24^ linked to the transmembrane and signalling domains of a CAR construct, namely CD28 TM followed by part of CD28 and CD3ζ intracellular domains^25^. To validate our TCR-CAR construct, we used two therapeutic TCRs: DMF5, a MELAN-A peptide specific TCR^26^ and Radium-1 TCR, a TCR targeting a TGF beta Receptor 2 (TGFbR2) frameshift mutation^27^. The first one had already been used in our previous studies^23, 24^ and shown to be very efficiently produced and extremely flexible in respect to the modifications it could accommodate. Radium-1 TCR has also been validated as sTCR, but only for binding to pMHC (our unpublished data). Both TCR-CARs were constructed and expressed but to our surprise DMF5 was less prone to accommodate this format. However, Radium-1 TCR-CAR was well detected and could also be seen in a CD3-free system such as the NK cell line, NK-92. Both TCR-CARs could redirect T cells and NK cells against their cognate pMHC, and we further showed that TCR-CAR could trigger target cell killing. Thus TCR-CAR might represent an alternative to redirect effector cells and render non-T cells pMHC-restricted, potentially opening the CAR targeting to the whole proteome.

## Results and Discussion

### Design of TCR-CAR

**Figure 1:**
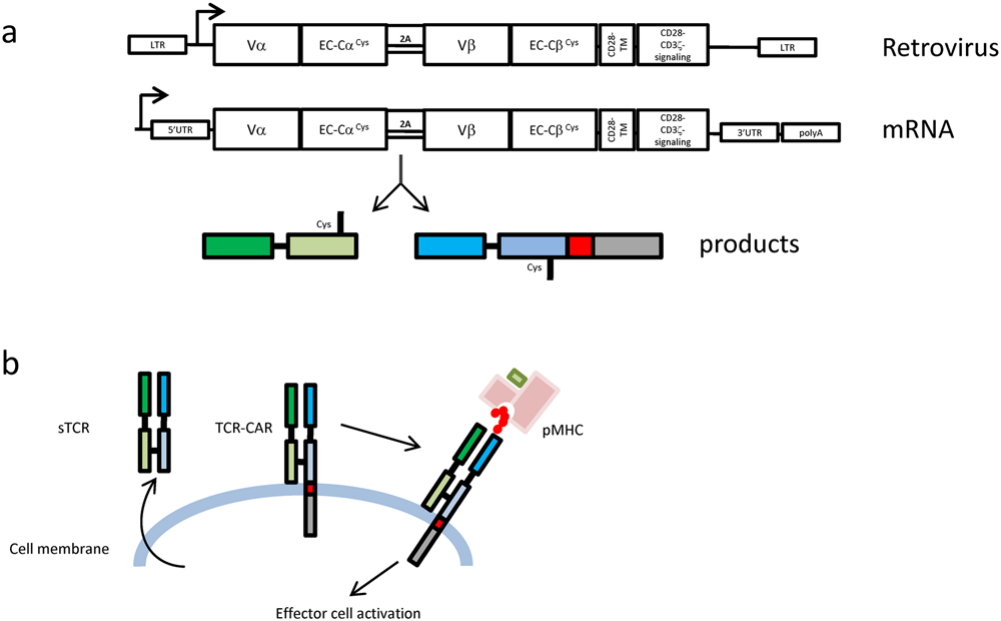
Design of the TCR-CAR constructs. (**a**) TCR-CAR gene design was based on the strategy previously used to produce soluble TCR (sTCR) in mammalian cells^24^: TCRα and β chain were truncated at the level of their TM region, cysteines were added on their constant domains and the two chains were linked by a 2A peptide sequence. The artificial STOP codon of the TCRβ chain sTCR was replaced by the transmembrane (TM) domain of CD28 followed by a second generation CAR signalling tail composed of CD28 and CD3ζ signalling domains. The expected product of the TCR-CAR coding sequence should be two separated proteins released in the ER at equimolar amounts. (**b**) sTCR was produce as a soluble protein which, probably following the vesicular secretion pathway, was released in the cellular medium (left). TCR-CAR is expected to be exported to the cell surface as an TCRα/β heterodimer. Correct folding should ensure specific binding to a peptide-MHC (pMHC) complex and signal transduction through CD28-CD3 signalling tail (right).

We have previously shown that one could efficiently express soluble TCR (sTCR) in Hek cells^24^. We obtained high yields (4 mg/L, unpublished data) of active material by taking advantage of the 2A-based expression system^28^. These results were *per se* not predictable as the release of the two separated soluble chains would not mechanically result in the formation of a stable molecule, as a likely outcome could be their degradation in the ER or never be sent to the plasma membrane. Since synthesis and export of sTCR generation were possible, we designed a related construct in which the TCRβ chain was fused to an artificial signalling domain similar to the one used for CARs (Fig. 1a): namely CD28 TM coding sequence followed by two signalling modules (CD28 and CD3ζ). In addition, a cysteine replacement was performed on the constant domain (C-domain) in order to increase the TCR dimer stability^16, 24^. This TCR-CAR cassette was subcloned into two different expression systems, namely MP71 retroviral vector and mRNA synthesis vector using the strategy published earlier^29^. We expected the protein product of the TCR-CAR construct to be exported to the plasma membrane like a receptor and that upon pMHC encounter, it would bind to its substrate and signal (Fig. 1b).

### Expression of two TCR-CAR

**Figure 2:**
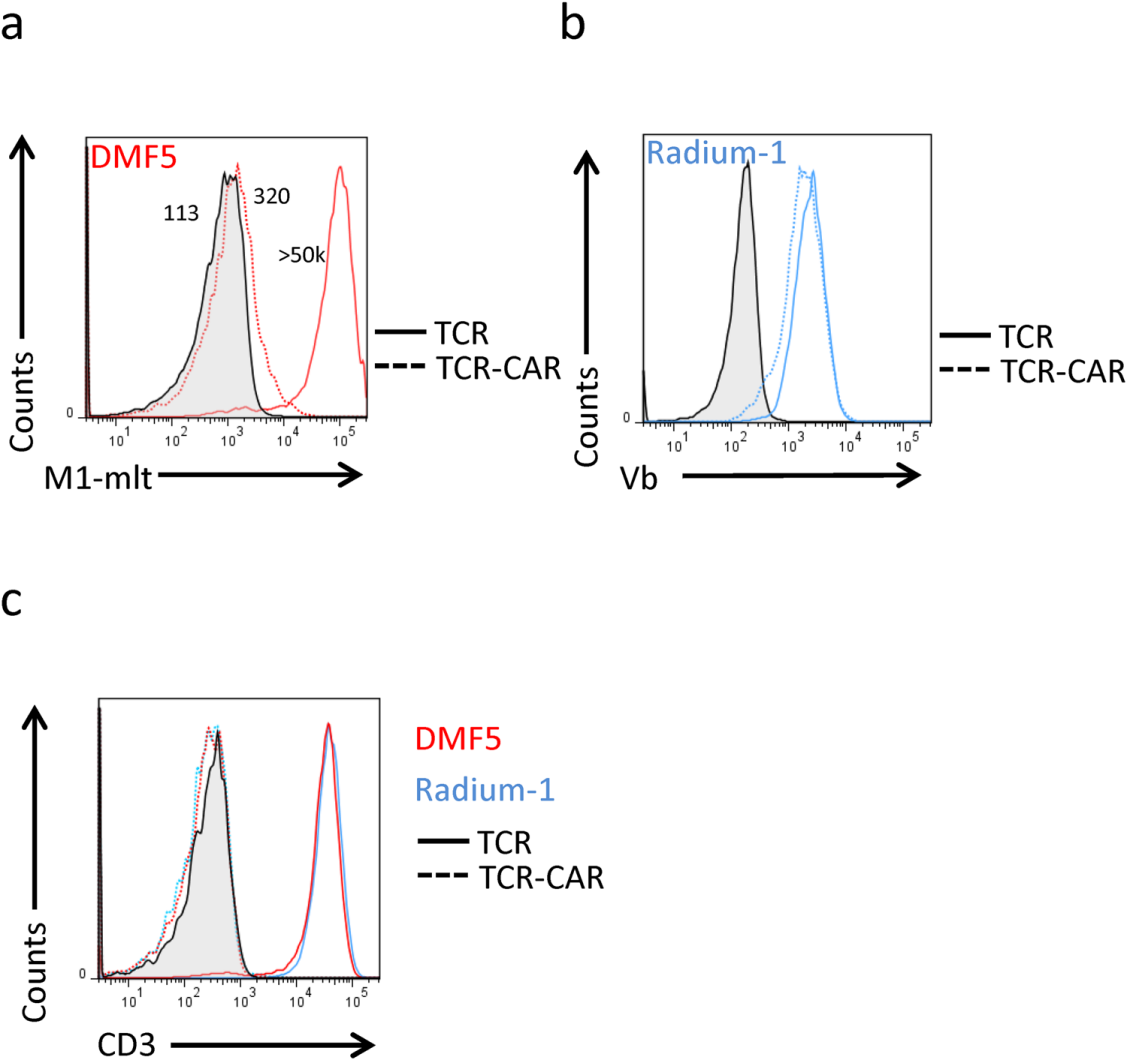
Membrane expression of TCR-CAR. (**a**) DMF5 TCR and TCR-CAR were expressed in J76 cell line. Forty-eight hours later, cells were stained with pMHC multimers of HLA-A2 in complex with MART-1 peptide (M1). Mock transduced cells (grey) were used as negative control, TCR and TCR-CAR (red, plain and dotted, respectively) are shown. Numbers specify MFI of the indicated staining. (**b**) Same as in (**a**) but Radium-1 TCR (plain) and TCR-CAR (dotted) were here expressed and J76 cells were stained with anti-Vb3 antibody (Vb). (**c**) The cells as in A (red) and B (blue) were stained with anti-CD3 antibody. As before, TCRs are shown as plain lines and TCR-CAR as dotted, and mock transduced is in grey. These are representative staining of similar experiments performed on different retroviral preparation of cells at least once.

We selected two MHC-Class I restricted TCRs, DMF5^26^ and Radium-1^27^ which are directed against the MART-1 peptide_26–35_ (EAAGIGILTV) and TGFbR2 frameshift neoantigen peptide_131–139_ (RLSSCVPVA)^30^, respectively. We first tested whether these constructs could be efficiently produced and sent to the plasma membrane. We compared expression of TCR-CAR with their corresponding full-length TCR in J76 cells which are TCR negative but become CD3 positive upon TCR expression^31^. DMF5 TCR and TCR-CAR were detected using a commercially available MART-1 dextramer (Fig. 2a). A weak expression of DMF5 TCR-CAR was detected, suggesting that it was either not well exported to the membrane or the protein was not stable when expressed in this format. Since there was no multimer available for Radium-1 TCR staining, we used an antibody against the Vbeta-chain of Radium-1 (anti-Vb3, Vb) to detect both constructs (Fig. 2b). Unlike what was observed with DMF5, Radium-1 TCR-CAR was expressed with a similar efficiency as its full-length TCR counterpart. On the other hand, DMF5 showed limited ability to bind the dextramer. Since the TCR-CAR proteins were expressed at the plasma membrane (Fig. 2a and b), we tested their ability to recruit CD3. As mentioned before, when a TCR is expressed in J76 cells, they become CD3 positive. However, CD3 staining showed that TCR-CAR did not interact with endogenous CD3 since J76 remained CD3 negative (Fig. 2c). This is in line with recent reports proposing an interaction between CD3 and TCR through the TM domains^32, 33^, which is not present in TCR-CAR and suggests that TCR-CAR acts independently of endogenous TCR signalling machinery probably due to the presence of CD28 TM domain. As for classical CAR constructs, we predicted that our construct would bypass the “CD3-block” due to the presence of CD28 TM domain. We also expected that TCR-CAR would not compete for the endogenous CD3. Radium-1 TCR and TCR-CAR were expressed at similar levels as detected by the specific Vb antibody. This suggests that Radium-1 TCR-CAR was probably well folded and likely composed of a Vb and a Va chains. On the other hand, DMF5 TCR-CAR was somehow less efficiently produced. This was surprising as this TCR was very stable when prepared as sTCR^23^. We also noticed that even sorted cells had a tendency to lose the MART-1-dextramer positive signal after several passages (data not shown), suggesting that DMF5 TCR-CAR might become detrimental to the cells expressing it at too high levels. It is worth mentioning that dextramer staining is a more stringent measure of expression than Vb staining since dextramer will only detect correctly folded and heterodimerized TCR chains. In conclusion, we observed that both TCR-CAR constructs were expressed at the membrane in J76 cells, but the level was somewhat lower than the full-length TCR. This could be due to a poor stability of the TCR-CAR construct. However, the lack of CD3 dependency represents a great advantage over classical overexpression of full-length TCR as it also means that TCR-CAR expression can be extended to other cells than T cells.

### T cells redirected by TCR-CAR

**Figure 3:**
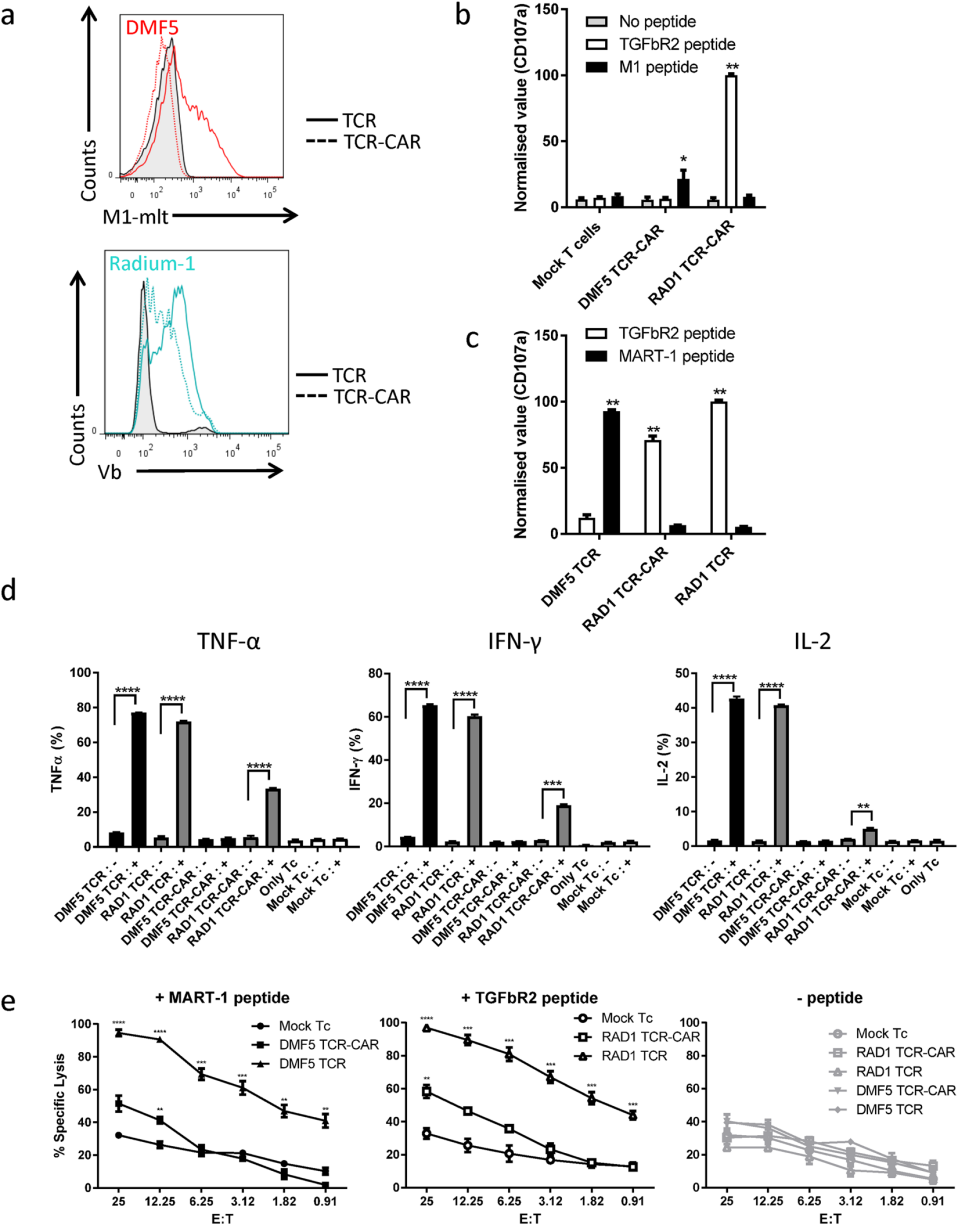
Functional activity of TCR-CAR. (**a**) Primary peripheral T cells isolated from a healthy donor were mock electroporated (tinted) or electroporated with mRNA encoding Radium-1 (blue) or DMF5 (red) constructs. After 12 hours, cells were stained with the indicated antibodies or multimer and analysed by flow cytometry. (**b**) The same cells were co-incubated 18 hours later with APCs loaded with the indicated peptides for 5 hours (grey = no peptide, white = TGFbR2 peptide and black = MART-1 peptide). The presence of the degranulation marker CD107a was performed to monitor T-cell stimulation. Data are representative of two separate experiments. Values were normalized to that of Radium-1 TCR CAR. Mean ± Standard error of the mean (SEM). N = 2. **P < 0.0001, *P < 0.001, 2-way ANOVA followed by Tukey’s multiple comparison test. (**c**) same as in (b) but here Radium-1 TCR-CAR was compared to the full-length Radium-1 TCR. DMF5 was included as a control for M1 loading. Mean ± SEM, N = 2. **P < 0.0001, 2-way ANOVA followed by Tukey’s multiple comparison test. (**d**) DMF5 (black) or Radium-1 (grey) TCR-CAR and full-length constructs expressing T cells were analyzed for the indicated cytokine response in the CD8 population. Target cells were loaded (+) or not (−) with the antigenic peptide and incubated with the T cells at an E:T ratio of 1:2. As a control mock transfected T cells (white) were tested in the same conditions or alone. Intracellular flow cytometry readings were collected after 6 hours of co-culture and % of positive cells were plotted. Mean ± SEM, N = 3. Unpaired t-test was used as statistical analysis. (**e**) same as in (**d**) where specific lysis of target cells loaded with the indicated peptide was analyzed. Transduced Tc with the indicated constructs were incubated at different E:T ratios with target and lysis was monitored by BLI cytotoxic assay. Luminescence readings were collected after 10 hours of co-culture. Mean ± SEM, N = 3. Unpaired t-test performed between indicated group and corresponding Mock group. Ranges for unpaired t-test were as follows *P < 0.05, **P < 0.01, ***P < 0.001, ****P < 0.00001.

The activity of a TCR can be evaluated in a functional assay in which target cells positive for the specific MHC loaded with the relevant peptide are used. We introduced TCR-CAR into primary T cells isolated from PBMC by mRNA electroporation^34^ and analysed the protein expression by flow cytometry (Fig. 3a). As shown both full-length TCRs were well expressed, TCR-CAR could be detected at a lower level than Radium-1 TCR, and DMF5 TCR-CAR could not be detected by multimer. Since multimer staining is not a highly sensitive method, the fact that DMF5 TCR-CAR was not detected by multimer does not mean that the protein was not present. We therefore tested whether primary T cells could be redirected against specific targets. Both the TCRs used here being HLA-A2 restricted, a myelogenous leukaemia cell line, K562, was transduced with HLA-A2 and used as APC. These cells pre-loaded with the indicated peptides were incubated with TCR-CAR redirected T cells. The T-cell activation was monitored five hours later by detecting the presence of the degranulation marker CD107a at the plasma membrane of the T cells. As shown (Fig. 3b), only the correct combination of pMHC was recognized by TCR-CAR. Mock electroporated T cells were used as a negative control and showed no stimulation. When DMF5 TCR-CAR was electroporated, a slight but significant activation was observed (Fig. 3b). This suggests that although DMF5 TCR-CAR expression in T cells was not detectable, some TCR-CAR activity could still be monitored. This is in agreement with our previous observation using mRNA electroporated conventional CAR T cells showing that even at protein levels not detectable by specific anti-CAR antibodies, the activity was present^34^. On the other hand, Radium-1 TCR-CAR showed a sustained pMHC-specific stimulation which matched the expression of the TCR-CAR detected by Vb3 staining (Fig. 3a). In order to study the level of stimulation TCR-CAR could induce, we repeated the experiment comparing Radium-1 TCR-CAR with full-length Radium-1 TCR and showed that both constructs had the capacity to trigger degranulation (Fig. 3c). Finally, we tested the ability of our constructs to redirect T cells and trigger cytokine release and target cell killing. In agreement with the CD107a expression, DMF5 TCR-CAR did not trigger cytokine release, but still managed to significantly kill peptide loaded APC (Fig. 3d and e, respectively). On the other hand, Radium-1 TCR-CAR redirected T cells were able to produce cytokines in a peptide-dependent manner and significantly kill target cells loaded with specific peptides (Fig. 3d and e). Radium-1 TCR performed more efficiently than TCR-CAR in both assays, but the TCR-CAR construct was functional, reaching statistical significance, suggesting that the receptor was potent. Taken together, our data show that if the TCR-CAR construct was expressed in primary T cells: (1) the recognition part of TCR-CAR maintained its specificity when fused to an artificial signalling domain and (2) the signalling part when fused to TCR could recruit endogenous signalling components to trigger degranulation and target cell killing. Although the values for both TCR-CARs were lower than the ones obtained with the full-length constructs, TCR-CARs were functional. This could largely be explained by the difference in expression between full-length TCR and TCR-CAR, but could also be influenced by other mechanisms such as non-optimal signalling for T cells when using target recognition domains from TCR rather than antibodies. This is important because TCR-CAR design can still be improved: antibody-based CARs have high affinity for their target, and tandem CD28-CD3ζ signalling modules might be sufficient for high affinity binding. Compared to CAR, TCR binding to pMHC is considered to be of relatively low affinity and we might therefore need to increase the number or the potency of the signalling boxes in the TCR-CAR construct in order to optimize the cytokine release and killing efficiency. TCR redirection of patient T cells can be improved by different means^35^, but influencing the signalling has rarely been exploited^36^. Indeed, it was previously reported by others that CD3 overexpression could improve TCR redirection potency^13^. This improvement probably resulted from the increased number of TCR molecules at the plasma membrane, including the endogenous TCR, which could result in increased mispairing, hence off-target effects. TCR-CAR did not compete for CD3 and signalled without being affected by the presence of endogenous TCRs.

### T-cell like redirection of NK cells

**Figure 4:**
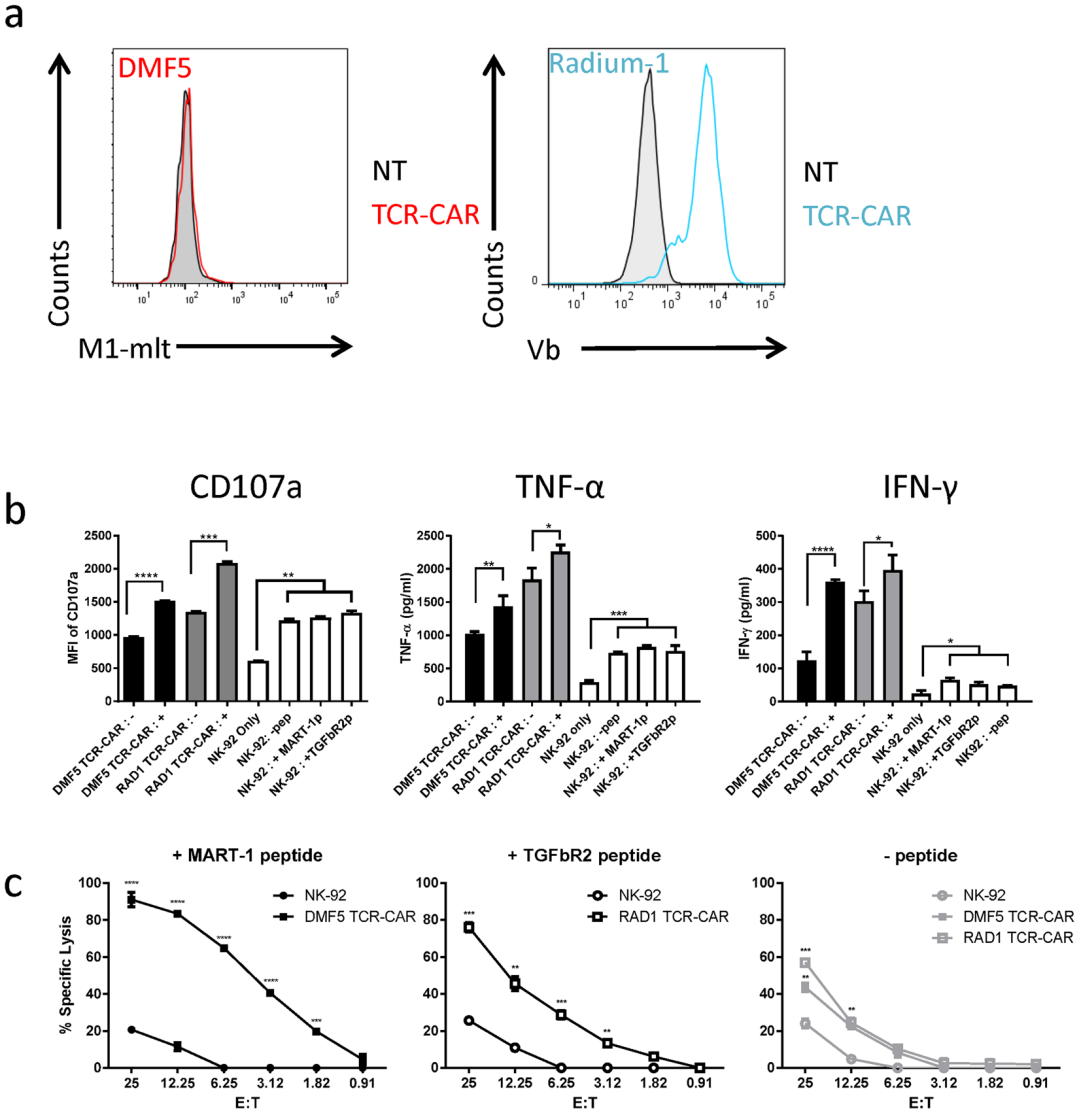
TCR-CAR can redirect NK-92 cells. (**a**) NK-92 cells were non transfected (grey) or transfected with DMF5 TCR-CAR TCR-CAR (red), Radium-1 (blue) and stained with specific antibody or multimer, respectively. Shown is a single staining representative of two separate stainings. (**b**) Stimulation of plain NK-92 cells (white) or transfected with TCR-CAR constructs (DMF5, black and Radium-1, grey) with Granta-519 loaded (+) or not (−) with the cognate peptide was performed for 6 hours at a E:T ratio of 1:2. Presence of CD107a was detected by flow cytometry and the MFI of the signal was plotted. Mean ± SEM, N = 3. Unpaired t-test was used as statistical analysis between indicated groups and similar trends were depicted as a group. For cytokines analysis, NK-92 cells transduced (black and grey) or not (white) were co-cultured with target cells loaded (+) or not (−) with the cognate peptide at 1:2 E:T ratio for 24 hours. Non transfected NK-92 cells alone (NK-92 only) were also tested. Supernatants from each condition were collected and presence of TNF-α and IFN-γ was performed with the Bio-Rad Bio-Plex 100 system. Measurements were made in triplicate from three separate supernatants per condition. Cytokine concentrations are shown in picograms per milliliter (pg/mL). Mean ± SEM, N = 3. Unpaired t-test was used as statistical analysis between indicated groups and similar trends were depicted as a group. (**c**) Specific lysis of target cells loaded with the indicated peptide (MART-1 peptide, black, TGFbR2 peptide, white, no peptide, grey) by plain NK-92 (circles) or NK-92 expressing TCR-CAR (squares) at different E:T ratios in a BLI cytotoxic assay. The specific lysis luminescence readings were collected after 10 hours of co-culture. Mean ± SEM, N = 3. Unpaired t-test performed between indicated group and corresponding non-transduced NK-92 group. Ranges for unpaired t-test were as follows *P < 0.05, **P < 0.01, ***P < 0.001, ****P < 0.00001.

As mentioned before, TCR-CAR carrying its own signalling units could potentially redirect other killer cells than T cells. We tested this by redirecting the non-T cell line, NK-92 which is a clinically approved natural killer cell line^37, 38^. We first confirmed that NK-92 cells were not able to express a full-length TCR by electroporating them with mRNA encoding either Radium-1 TCR or Radium-1 TCR-CAR and staining them with an anti-Vb3 antibody (Supplementary Fig. S1). As shown, only the TCR-CAR construct could be detected at the cell surface of NK-92, whereas in the same conditions the T-cell line J76 expressed both constructs. Therefore, NK-92 cells were not able to express a full-length TCR at their cell surface. We then retrovirally transduced NK-92 cells with our TCR-CAR constructs and after two rounds of spinoculation we obtained a large population of Vb3-positive NK-92 cells, indicating that the TCR-CAR could stably be expressed, folded and targeted at the surface of a non-T cell line (Fig. 4a). In contrast, the DMF5 TCR-CAR could not be detected using multimer (Fig. 4a), but as mentioned before, lack of multimer binding does not necessarily imply that the protein is not present. We then performed functional assays in order to study the activity of TCR-CAR in a non-T cell effector cell line, NK-92. To this end we had to change the target cells since K562 are commonly used as NK cell targets, and could generate elevated background responses, thus reducing the impact of the TCR stimulation. When we proceeded to a functional assay to detect CD107a, we noticed that CD107a signal was high in the presence of different target cells (data not shown). This was probably due to NK-92 natural reactivity against tumour cell lines. We tested different HLA-A2 positive cell lines and selected the “most resistant” to NK-92 in a killing assay and found out that the B cell lymphoma cell line Granta-519, an HLA-A2 positive transformed mantle cell lymphoma, showed the lowest reactivity. We thus co-incubated them with NK-92-TCR-CAR after loading or not with the relevant peptide and studied cytokine release and killing activity of redirected NK-92 cells (Fig. 4b and c, respectively). We first looked at the degranulation marker CD107a expression and different cytokines (IFN-γ, TNF-α) upon target stimulation. As shown, NK-92 incubated with Granta-519 was stimulated (Fig. 4b, white columns) compared with NK-92 alone. However, the stimulation was significantly increased when TCR-CARs were expressed in NK-92 in the presence of peptide-loaded targets. Thus both TCR-CARs were expressed in NK-92 cells, and even if not detectable, were able to trigger pMHC-specific cytokine release. The overall background was higher in TCR-CAR expressing cells, suggesting that the constructs were functional and possibly gave some activation of NK-92 cells without binding their target. We next tested the capacity of NK-92 and NK-92-TCR-CAR cells to kill target cells (Fig. 4c). The enhanced killing of peptide loaded cells was observed even at low E:T ratio, suggesting that the killing was sensitive. In addition, even if at high E:T ratio NK-92 cells could kill target cells independently of the pMHC presence (Fig. 4c circles, maximum killing in the three conditions is 30% at E:T 1:25), TCR-CAR expression dramatically improved the recognition and the killing of the targets. Interestingly, although not detectable by multimer staining, DMF5 TCR-CAR modified NK-92 cells became much more potent killers of MART-1 peptide loaded tumour cells than non-modified NK-92 cells, suggesting that this TCR-CAR, even at low expression, was active and specific. We also performed killing using unloaded Granta-519 as targets (Fig. 4c, right panel). This showed that despite specific TCR-dependent killing in the presence of peptide, killing of non loaded targets was observed to a higher degree and in an E:T ratio dependent manner by the TCR-CAR expressing NK-92 cells compared to NK-92 cells. This is in agreement with the increased basal cytokine release in NK-92 cells expressing TCR-CAR (Fig. 4b, black and grey columns) and suggests that the presence of TCR-CAR somehow activated NK-92 cells. As mentioned before, the TCR-CAR presented herein is a first generation and the use of cell type-specific signalling boxes might improve the functional outcome and decrease the background. The backbone of the TCR-CAR construct presented in the present paper is a single chain TCR-based construct carrying its own signalling domain, and in this perspective TCR-CAR accomplished its mission: it was able to conserve the original TCR specificity and was independent of the endogenous early signalling components. Furthermore, knowing that in clinical settings, NK-92 cells are irradiated prior to infusion, it might be an advantage to have them pre-activated. In conclusion, TCR-CARs were able to redirect cells other than T cells to generate a TCR-dependent killing. Collectively, these data show that TCR-CAR expands the TCR expression spectrum to cells other than T cells. NK-92 cells have previously been exploited either naked or redirected with CAR. Tumour-specific surface antigen targets being scarce, TCR-CAR redirection is opening new opportunities for targeting of NK cell-based adoptive transfer.

## Methods

### Cell lines, Media, Chemicals and Peptides

T cells were obtained from buffy coats from healthy blood donors from the blood bank (Ullevål hospital, Oslo, Norway). Informed consent was obtained from all subjects. The ethical approval of project no 2013/624–15 of the study was given by the Ethical Committee of the South-Eastern Norway Regional Health Authority and the study was carried out in accordance with institutional and ethical guidelines. J76^31^ (a kind gift from M. Heemskerk, Leiden University Medical Center, The Nederlands) were maintained in RPMI (PAA, Paschung, Austria) supplemented with 10% HyClone FCS (HyClone, Logan, UT, USA) and gentamicin (50 µg/mL) K562 (ATCC, CCL-243), Granta-519 (DSMZ, ACC 342) and T2 cells were maintained in the same medium. The packaging cells were the modified Human Embryonic Kidney cells-293, Hek-Phoenix (Hek-P) and they were grown in DMEM (PAA) with 10% FCS. T cells were grown in CellGro DC medium (CellGenix, Freiburg, Germany) supplemented with 5% heat-inactivated human serum (Trina Bioreactives AG, Nänikon, Switzerland), 1.25 mg/mL N-acetylcysteine (Mucomyst 200 mg/mL, AstraZeneca AS, London, UK), 0.01 M HEPES (Life Technologies, Norway) gentamycin 0.05 mg/mL (Garamycin, Schering-Plough Europe, Belgium). NK-92 cells were cultured and maintained in X-Vivo 10 medium supplemented with 5% heat-inactivated HS and 500 IU/mL IL-2. The TGFbR2 frameshift peptide_131–139_, RLSSCVPVA was provided by Norsk Hydro ASA, (Porsgrunn, Norway). The MART-1 peptide_26-35_ EAAGIGILTV was manufactured by ProImmune Ltd (Oxford, UK) and MART-1 dextramer was from Immudex (Copenhagen, Denmark).

### DNA Constructs

The TM and cytosolic domain from the CAR^25^ domain was added on to the previously described soluble TCR^24^ by overlapping PCR by using the following primers: CAR template (5′-3′) forward gggtagagcagactgtggtaaattttgggtgctggtggtgg (1), reverse ctcgagttagcgaggaggcagggcctgcatgtgaag (2), sTCR template forward (Radium1) caccatgaagaggatat (3), (DMF5) caccatgatgaaatcct (4), reverse ccaccaccagcacccaaaatttaccacagtctgctctaccc (5). The two PCR products were subsequently combined into the TCR-CAR using the following primer pair (3) and (2) for Radium-1 and (4) and 3(2) for DMF5. The final PCR product was cloned into pENTR (Themofisher, Waltham, MA, USA). Sequence verified constructs were recombined into a Gateway-modified pMP71 (retroviral vector) or pCIpA102 (mRNA synthesis construct) as described in ref. 29. TCR expression constructs used here were described in refs 27 and 29 for DMF5 and Radium-1, respectively. The HLA-A2 construct was already described in ref. 39, Addgene (Plasmid #85162).

### *In vitro* mRNA transcription

The *in vitro* mRNA synthesis was performed essentially as previously described^40^. Anti-Reverse Cap Analog (Trilink Biotechnologies Inc., San Diego, CA, USA) were used to cap the RNA. The mRNA quality was assessed by agarose gel electrophoresis and Nanodrop (Thermo Fisher Scientific).

### *In vitro* expansion and electroporation of T cells

T cells from healthy donors were expanded using a protocol adapted for GMP production of T cells employing Dynabeads CD3/CD28 as described in ref. 34. In brief, PBMCs were isolated from buffy coats by density gradient centrifugation and cultured with Dynabeads (Dynabeads® *ClinExVivo*™ CD3/CD28, ThermoFischer, Oslo, Norway) at a 3:1 ratio in complete CellGro DC Medium with 100 U/mL recombinant human interleukin-2 (IL-2) (Proleukin, Prometheus Laboratories Inc., San Diego, CA, USA) for 10 days. The cells were frozen and aliquots were thawed and rested in complete medium before transfection. Expanded T cells were washed twice and resuspended in CellGro DC medium (CellGenix GmbH) to 70 × 10^6^ cells/mL. The mRNA was mixed with the cell suspension at 100 μg/mL, and electroporated in a 4-mm gap cuvette at 500 V and 2 ms using a BTX 830 Square Wave Electroporator (BTX Technologies Inc., Hawthorne, NY, USA). Immediately after transfection, T cells were transferred to complete culture medium at 37 °C in 5% CO_2_ overnight.

### Retroviral transduction of NK-92 and preparation of K562 (HLA-A2)

Viral particles were produced as described in ref. 29 and were used to transduce NK-92 and K562 cells as follows: Spinoculation was performed with 1 Volume of retroviral supernatant mixed with 1 Volume of cells (0.3 M/mL) in a 12-well (2 mL final) or a 24-Well (1 mL final) non-treated plate (Nunc A/S, Roskilde, Denmark) pre-coated with retronectin (50 µg/mL, Takara Bio. Inc., Shiga, Japan). NK-92 cells were spinoculated twice at 32 °C at 750X g for 60 min. Cells were then harvested with PBS-EDTA (0.5 mM) and grown in their regular medium.

### Functional Assay and Flow Cytometry

K562 (HLA-A2) or Granta-519 cells were loaded with peptide overnight at 37 °C in a 5% CO_2_ incubator. Effector cells were stimulated with target cells at an effector-to-target (E:T) ratio of 1:2 for 5 hours at the same conditions as above. Conjugated CD107a was added to the cells prior to incubation. Irrelevant or no peptide served as a negative control.

The following antibodies were used: Vβ3- FITC (Beckman Coulter-Immunotech SAS, France), CD3-eFluor450, CD56- eFluor, CD107a-PE-Cy5, TNFα-PE (BD Biosciences, USA), IL2-APC, IFNγ-FITC (eBiosciences, ThermoFischer). Cells were washed in flow buffer (FB, phosphate buffered saline (PBS) with 2% human bovine serum albumin (BSA) and 0.5 µM EDTA). For dextramer and antibody staining, cells were incubated for 30 minutes at room temperature (RT) with the recommended dilution in FB. If fixed, cells were incubated in FB containing 1% paraformaldehyde. For intracellular staining Perm/Wash Buffer was used (BD Biosciences) according to manufacturer’s protocol. All antibodies were purchased from eBioscience, USA, except where noted. Cells were acquired on a BD FACSCanto II flow cytometer and the data analyzed using FlowJo software (Treestar Inc., Ashland, OR, USA). Plotting and statistical analysis were performed using GraphPad prism software (La Jolla, CA, USA).

### Bioluminescence-based Cytotoxicity Assay

Luciferase-expressing tumor cells were counted and resuspended at a concentration of 3 × 10^5^ cells/mL. Xenolight D-Luciferin potassium salt (75 µg/mL; Perkin Elmer, Oslo, Norway) was added to tumor cells which were placed in 96-well white round bottomed plates at 100 µL cell suspension/well in triplicates. Subsequently, effector cells were added as indicated effector-to-target (E:T) ratios. In order to determine baseline cell death and maximal killing capacity, three wells were left with only target cells and another three with target cells in 1% Triton^™^ X-100 (Sigma-Aldrich). Cells incubated at 37 °C for 2 hours. Bioluminescence (BLI) was measured with a luminometer (VICTOR Multilabel Plate Reader, Perkin Elmer) as relative light units (RLU). Target cells that were incubated without any effector cells were used to determine baseline spontaneous death RLU in each time point. Triplicate wells were averaged and lysis percentage was calculated using following equation: % specific lysis = 100x(spontaneous cell death RLU- sample RLU)/(spontaneous death RLU – maximal killing RLU). Plotting and statistical analysis were performed using GraphPad prism software.

### Cytokine Measurements

Cytokines released from transduced or non-transduced NK-92 cells incubated with Granta-519 cells were collected after 24 hours of co-culture. Cytokines in supernatants were measured by using the Bio-Plex Pro^TM^ Human Cytokine 17-plex Assay (Bio-Rad Laboratories, Hercules, CA, USA) according to manufacturer’s protocol on a Bio-Rad Bio-Plex 100 system. Plotting and statistical analyses were performed using GraphPad prism software.

### Data Availability

The datasets generated during and/or analysed during the current study are available from the corresponding author on reasonable request.

## Electronic supplementary material

Expression analysis of Radium-1 TCR and TCR-CAR
