# Supplementary material for: A TCR-based Chimeric Antigen Receptor
Source: Sci Rep. 2017 Sep 6;7:10713. doi: 10.1038/s41598-017-11126-y (PMC5587706; doi:10.1038/s41598-017-11126-y)
Supplement: Supplementary file 1 — Expression analysis of Radium-1 TCR and TCR-CAR [file 41598_2017_11126_MOESM1_ESM.pdf]

Supplementary Figure

A TCR-based Chimeric Antigen Receptor

Even Walseng, Hakan Köksal, Ibrahim M. Sektioglu, Anne Fåne, Gjertrud Skorstad, Gunnar Kvalheim, Gustav Gaudernack, Else Marit Inderberg, Sébastien Wälchli

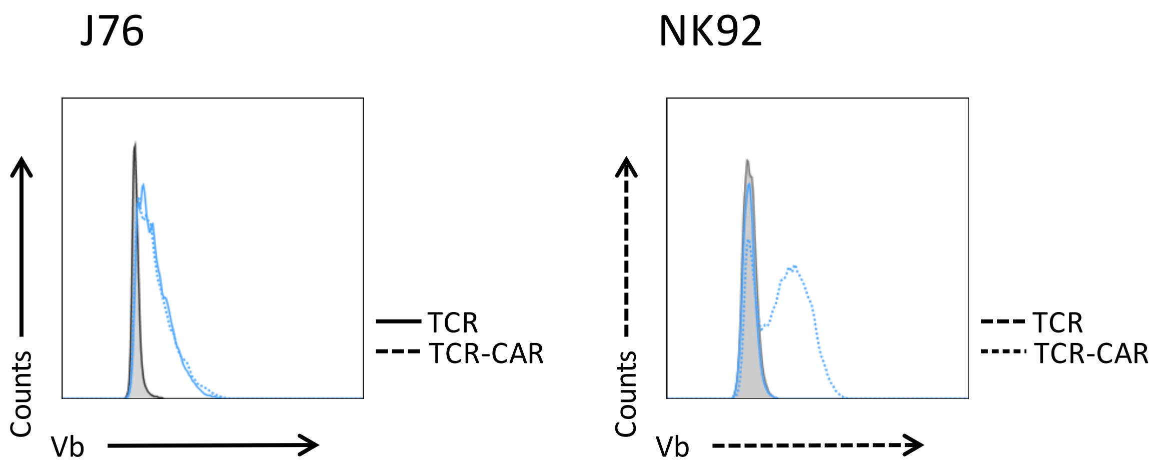

Supplementary Figure S1: Expression analysis of Radium-1 TCR and TCR-CAR J76 and NK-92 cells were electroporated with water (grey), Radium-1 TCR mRNA (solid line) or Radium-1 TCR-CAR mRNA (dashed line). Expression of Radium-1 was detected using anti-Vb3 antibody (Vb) and analysed by flow cytometry 18 hours after electroporation. These data are representative of two separated experiments.
